# Wastewater as Sentinel for Emerging Viral Diseases in Livestock: A Systematic Review

**DOI:** 10.3390/v18030385

**Published:** 2026-03-19

**Authors:** Mishuk Shaha, Ashutosh Das, Joyshri Saha, Md. Mizanur Rahaman, Mukta Das Gupta, Saranika Talukder, Subir Sarker

**Affiliations:** 1 Department of Genetics and Animal Breeding, Faculty of Veterinary Medicine, Chattogram Veterinary and Animal Sciences University, Chattogram 4202, Bangladesh; drmishukshaha38@gmail.com (M.S.); ashutosh.das@cvasu.ac.bd (A.D.); 2 Department of Economics, Noakhali Government College, Noakhali 3800, Bangladesh; joyshrijoya1@gmail.com; 3 Biomedical Sciences and Molecular Biology, College of Medicine and Dentistry, James Cook University, Townsville, QLD 4811, Australia; mdmizanur.rahaman@my.jcu.edu.au; 4 Australian Institute of Tropical Health and Medicine, James Cook University, Townsville, QLD 4811, Australia; 5 Department of Microbiology and Veterinary Public Health, Faculty of Veterinary Medicine, Chattogram Veterinary and Animal Sciences University, Chattogram 4202, Bangladesh; mukta_as@yahoo.com; 6 College of Science and Engineering, James Cook University, Townsville, QLD 4811, Australia; saranika.talukder@jcu.edu.au

**Keywords:** livestock, wastewater-based surveillance, emerging viral pathogens, environmental drivers, zoonotic spillover, One Health

## Abstract

The accelerating frequency of emerging infectious diseases (EIDs) in livestock poses a significant threat to global food security, as well as to animal and public health. While wastewater-based surveillance (WBS) has advanced significantly for human health surveillance, its application to livestock production systems remains fragmented and lacks standardization. This review synthesizes current evidence on livestock wastewater-based surveillance (L-WBS) as an early-warning sentinel for emerging viral pathogens, evaluating their dynamics, economic impacts, biosecurity measures, and One Health implications. Existing studies demonstrate that L-WBS effectively detects emerging viral pathogens in agricultural effluent, swine manure, and municipal wastewater systems serving livestock regions, frequently preceding clinical outbreak recognition. We further conceptualized a multifactorial framework linking environmental drivers such as climate and ecological disruption and agricultural intensification to pathogen emergence dynamics. Economic assessments show substantial direct losses (approximately US$ 950 per H5N1-infected dairy cow and US$ 25.9 billion in African swine fever virus (ASFV)-related damages across China) alongside indirect costs from biosecurity implementation, workforce disruption, and supply-chain instability. We recommend prioritizing methodological standardization through unified sampling and extraction protocols, integration of next-generation sequencing for genomic surveillance, and cross-sectoral policy frameworks to operationalize L-WBS as a global early-warning infrastructure for mitigating zoonotic spillover and livestock-dependent community resilience.

## 1. Introduction

The 21st century is defined, in part, by the accelerating frequency and impact of emerging and re-emerging infectious diseases. This phenomenon is not merely a public health challenge but a profound, systemic threat to global socioeconomic stability and, most importantly, to the integrity of the food systems that support the lives of 11 billion people, placing the livestock sector at the epicenter of this vulnerability [1]. Intensive farming systems—characterized by dense animal populations, extensive global trade in animal products, and continuous genetic variation within pathogen reservoirs—create a hyper-efficient environment for the emergence, evolution, and spread of disease [2]. The consequences of such outbreaks are catastrophic: beyond the immediate economic devastation caused by mass culling, trade restrictions, and production losses, there lies the ever-present, insidious risk of zoonotic spillover. Historically, the most devastating human pandemics, from avian influenza to severe acute respiratory syndrome coronavirus 2 (SARS-CoV-2), have their origins in the animal–human interface [3], making the health status of global livestock populations a direct proxy for global public health security.

To address systemic gaps in the current surveillance framework, a comprehensive and systemic review of L-WBS is urgently needed [4,5]. Although WBS for human health has made significant advances, particularly during the COVID-19 pandemic, most notably through early detection of SARS-CoV-2 variants, with real-time tracking of infection trends at the community level and quantification of viral loads weeks before clinical case surges, the application and adaptation of similar approaches to livestock systems are still conceptually underdeveloped and highly fragmented [6,7,8,9,10]. L-WBS lags due to unique challenges including diverse and complex wastewater matrices from animal housing, lack of standardized sampling protocols tailored to farm-scale variability, limited investment in pathogen-specific molecular assays for veterinary targets, and insufficient integration with existing livestock health reporting systems, resulting in sparse data and poor scalability across global farming contexts [11,12]. EIDs in livestock primarily stem from intensified agricultural practices, land-use changes such as deforestation that increase wildlife–livestock–human interfaces, and the global spread of industrial farming systems confining mixed animal species under high-density conditions [13,14]. These drivers facilitate pathogen spillover from wildlife reservoirs and genetic mutations, amplifying the issue through antibiotic overuse and inadequate biosecurity, positioning the livestock sector at the epicenter of economic vulnerability and public health threats. Following initial infection, transmission intensifies through airborne spread in enclosed barns, contaminated feed and water systems, transit of asymptomatic carriers across trade networks, and persistent environmental contamination in manure lagoons, often eluding early clinical detection [14,15].

Traditional surveillance methods, which mostly rely on targeted biological samples and clinical reporting, frequently experience critical delays [2,16] in identifying and responding to outbreaks and are unable to monitor asymptomatic or subclinical infections. In contrast, recent WBS innovations, such as droplet digital PCR (ddPCR), biosensor integration, mobile-linked microfluidics, AI-normalized flow proxies, CrAss-like phage tracking, and environmental metagenomic sequencing, complement and strengthen conventional systems by enhancing sensitivity, decentralization, and multi-pathogen capacity for emerging livestock threats [9,17]. Although human WBS has transformed early detection and public health intelligence, the scientific literature on L-WBS is highly fragmented, lacks standardization, and is predominantly focused on endemic pathogens or geographically restricted diseases [18,19]. In this review, ‘emerging viral pathogens’ refers to viruses that have newly appeared in livestock populations or are demonstrating emergence through rapid increases in incidence, novel geographic expansion, the appearance of new genetic variants/lineages, or cross-species spillover into new host populations (including events involving viruses that may be endemic elsewhere). Consequently, while pathogens like H5N1 or ASFV may be endemic in certain regions, their inclusion in this framework reflects their ongoing capacity for novel emergence, whereas routine monitoring of static, established endemic viruses falls outside the scope of this study.

The current literature on environmental pathogen surveillance exhibits three significant methodological limitations. Firstly, the lack of standardized protocols for pathogen sampling and molecular detection impedes the comparability of results across independent investigations and presents notable challenges for effective regulatory adoption. Secondly, most existing research does not adequately address emerging livestock pathogens; rather, the literature is geographically biased and primarily focuses on antibiotic resistance genes. Thirdly, the epidemiological utility of wastewater-derived pathogen markers as reliable early-warning indicators remains uncertain, including the quantitative and temporal correspondence between these markers; actual herd-level disease prevalence requires further investigation to validate their effectiveness relative to traditional surveillance strategies.

Given these knowledge gaps and the foundational role of surveillance in advancing One Health approaches, a comprehensive, authoritative study is needed to chart the terrain of L-WBS application, methodology, and epidemiological validity [20,21]. This study places livestock surveillance within the broader One Health paradigm, consistent with the World Organization for Animal Health’s (WOAH) definition of surveillance as “the systematic ongoing collection, collation, and analysis of information related to animal health and the timely dissemination of information so that policy makers can take action” [22]. Therefore, this review aims to elucidate current methodologies, conceptual advancements, and significant gaps in global frameworks for EID surveillance and risk assessment to overcome operational and epistemological barriers hindering cross-sectoral EID management and early-warning systems.

## 2. Materials and Methods

This systematic review studies emerging viral pathogens affecting livestock, assessing their economic impacts and surveillance to support a One Health approach. We followed the Preferred Reporting Items for Systematic Reviews and Meta-Analyses (PRISMA) 2020 protocol [23] to ensure a standardized and transparent reporting process (Supplementary Table S1).

### 2.1. Information Sources and Search Strategy

We systematically searched four major electronic databases, namely Google Scholar, PubMed, Scopus, and Web of Science [24], for peer-reviewed articles that were published up to 30 November 2025. The search strategy was built using a combination of controlled vocabulary, such as Medical Subject Headings [MeSH] [25] and extensive free-text terms and synonyms to maximize retrieval across the different indexing systems. Key search terms encompassed the exposure/methodology and the pathogens of interest, including “wastewater,” “sewage,” “wastewater-based epidemiology,” “environmental surveillance,” “wastewater monitoring,” “environmental monitoring,” “emerging diseases,” “livestock viral pathogens,” “livestock” “feces,” and “disease.” We incorporated synonyms and alternative spellings (e.g., “waste-water,” “sewer,” “environmental epidemiology”) to maximize retrieval sensitivity. To accommodate the distinct search interfaces across the platforms, the core search string was systematically adapted using database-specific syntax and Boolean operators. For instance, we used [MeSH] terms and [Title/Abstract] field tags in PubMed; TITLE-ABS-KEY operators in Scopus; and TS = (Topic) field tags in Web of Science. Following the execution of the searches, all retrieved citations were compiled and imported into the EndNote (version 21.3). Deduplication was then systematically performed using EndNote’s automated duplicate identification function, followed immediately by a rigorous manual verification step by the reviewers to remove any remaining hidden duplicates and ensure strict data integrity prior to the screening phase.

### 2.2. Inclusion and Exclusion Criteria

#### 2.2.1. Inclusion Criteria

Given the expedited timeline and focused nature of this review, a modified Population, Intervention, Control, and Outcomes (PICO) framework guided [26] the development and refinement of the search strategy, ensuring precise alignment with the research question’s boundaries, the core search will be structured as follows: (Block 1: Livestock/Animal) AND (Block 2: Surveillance) AND (Block 3: Pathogen Status) [27]. Studies were included if they met the following criteria:

- Block 1: Population: Focused on studies relevant to livestock and domestic animals, excluding general environmental or human studies.
- Block 2: Intervention: Centered on the environmental matrix of interest, specifically WBS and its role in the management or monitoring of emerging pathogens.
- Block 3: Outcomes: Focused on the emerging nature of the diseases and the significance of WBS in the management or monitoring of emerging pathogens (including terms like ‘Viral Pathogens’, ‘Virus’, ‘Disease’, ‘Sewage’, ‘Feces’, and ‘Sewer’), which is a key distinguishing feature of our review.

#### 2.2.2. Exclusion Criteria

Studies were systematically excluded if they met any of the following criteria, ensuring only primary research directly addressing the review’s scope is included.

##### Host and Scope

Human-exclusive and generalized urban focus: To eliminate conceptual confusion between human and animal surveillance, studies analyzing wastewater collected from urban or municipal sewage treatment plants were strictly excluded unless they specifically monitored distinct agricultural, slaughterhouse, meat-processing, or abattoir effluent lines. General urban sewage studies reflecting mixed or exclusively human domestic waste were excluded to delineate L-WBS boundaries strictly. Irrelevant host species: Studies that focused on companion animals that lack documented transmission risk to livestock or agricultural systems were excluded.

##### Monitoring Matrix and Data

Non-wastewater matrices: Studies used clinical samples and non-effluent environmental media (e.g., air, soil). The sample matrix must be wastewater, sewage, lagoon, manure, or agricultural effluent.

##### Disease Focus and Agent Type

- Non-infectious agents: Studies focused exclusively on chemical contaminants, illicit drugs, or pharmaceuticals.
- AMR only: Studies measuring only Antimicrobial Resistance (AMR) genes without concurrent identification or quantification of a target infectious livestock pathogen.
- Endemic focus: Studies focusing on routine monitoring of established, non-emerging, non-zoonotic endemic diseases.

##### Publication Type

- Language: Articles published in a language other than English.

### 2.3. Risk of Bias in Individual Studies

**Figure 1:**
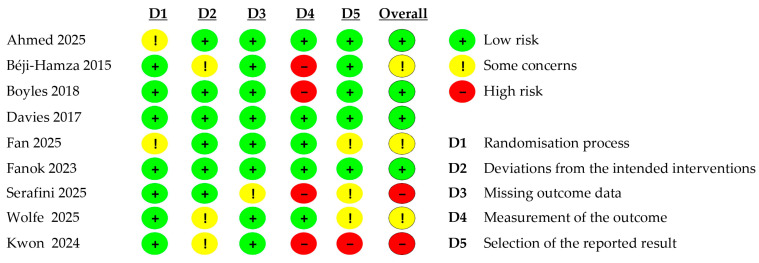
Risk-of-bias assessment of included studies using the Cochrane RoB 2 tool (traffic-light plot) [28,29,30,31,32,33,34,35,36]. +, low risk of bias; −, high risk of bias; !, some risk of bias (details can be seen Supplementary Table S2).

To assess the methodological quality and reliability of the included studies, we employed the Cochrane risk-of-bias assessment criteria, specifically the RoB 2 tool (revised version, 22 August 2019). This standardized tool evaluates bias across five critical domains: (D1) randomisation process, (D2) deviations from intended interventions, (D3) missing outcome data, (D4) measurement of the outcome, and (D5) selection of the reported result. Each domain was assessed through a series of signaling questions, with responses categorized as “low risk of bias” (indicated by a green ‘+’ symbol), “high risk of bias” (indicated by a red ‘−’ symbol), or “some concerns” (indicated by a yellow ‘!’ symbol) (Figure 1).

Three independent reviewers (M.S. and M.M.R.) conducted the bias assessments for all included studies, with disagreements resolved through discussion or consultation with a third reviewer (M.D.G). The overall risk-of-bias judgment for each study was determined following the RoB2 algorithm: studies were classified as “low risk” if all domains were rated as low risk, “some concerns” if at least one domain raised some concerns without any domain being at high risk, or “high risk” if one or more domains were judged to be at high risk.

### 2.4. Study Selection Process

**Figure 2:**
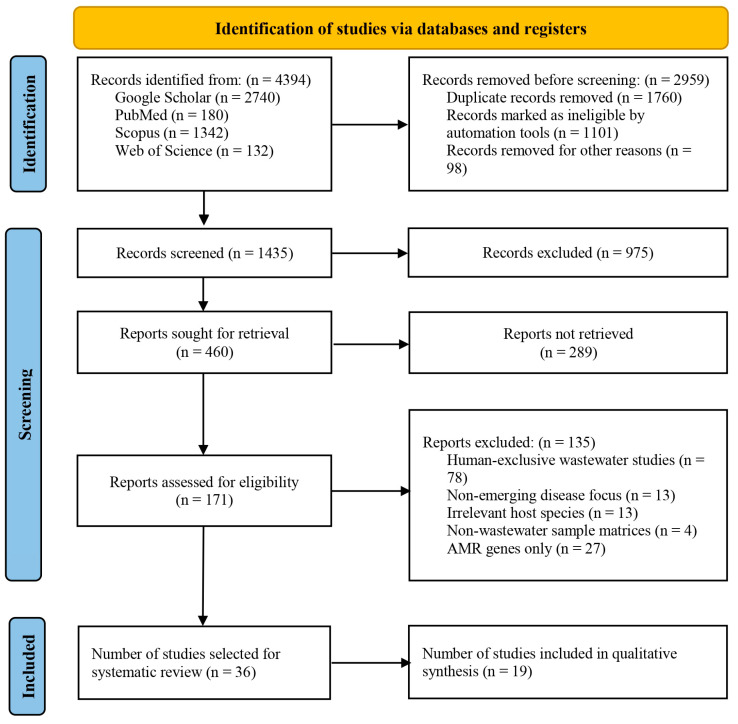
PRISMA flow diagram based on data extraction.

A total of 4394 records were identified through database searching. After removing duplicates and ineligible records, 1435 titles and abstracts were screened. Of these, 460 full-text articles were sought, of which 289 were unavailable or not retrieved. Of the 171 articles assessed for eligibility, 135 were excluded for failing to meet the inclusion criteria. Ultimately, 36 studies were selected for the systematic review, of which 19 were included in the qualitative synthesis. The whole study selection process is illustrated in a PRISMA flow diagram (Figure 2).

### 2.5. Data Extraction and Characteristics of Sources

Data were extracted using a standardized form, focusing on virus type, host species, disease mechanisms, environmental interactions, economic impacts, and biosecurity measures. Special attention was paid to emerging viral pathogens and mitigation strategies under changing environmental conditions.

## 3. Results and Discussion

### 3.1. Quality Assessment and Risk-of-Bias Interpretation

The risk-of-bias assessment using the Cochrane RoB 2 tool indicated that most included studies had a ‘low’ to ‘moderate’ risk of bias (Figure 1). Across observational and cross-sectional designs, primary concerns arose in the ‘deviations from intended interventions’ and ‘measurement of the outcome’ domains. These biases stem from inherent environmental sampling challenges, including inconsistent livestock wastewater matrices (e.g., fluctuating manure solid-to-liquid ratios) and a lack of standardized viral recovery controls. Consequently, while qualitative detection (presence/absence) of notable pathogens such as H5N1 and ASFV in the reviewed studies is robust, quantitative viral load metrics and their direct temporal correlations with clinical outbreaks should be interpreted cautiously. Ultimately, these methodological biases reinforce our conclusion that standardizing matrix-specific extraction protocols is essential for L-WBS to become a reliable, quantitative early-warning system.

### 3.2. Diversity of Emerging Livestock Pathogens and Detection Challenges

As a consequence of globalization and climate change, both animal and public health are currently facing a ground-breaking impact of emerging and re-emerging animal diseases and zoonoses worldwide. An emerging disease is a novel infection that has recently appeared in a population for the first time or is rapidly increasing in its geographical range or incidence, causing significant morbidity or mortality in animals or humans. A completely new pathogen or evolution, a mutated version of an existing one, or a known infection spreading to a new area can cause it. In animals, these diseases are often significant because they have the potential to disrupt ecosystems, impact food security, or jump to humans as zoonotic infections. A known or endemic disease is considered re-emerging if it shifts its geographic range, expands its host range, or increases its incidence.

Livestock effluent and wastewater have become influential environmental reservoirs that contribute to the transmission and emergence of infectious disease. These waste streams often contain a variety of pathogens, including virus (norovirus, rotavirus adenovirus, coronavirus, astrovirus, etc.), bacteria (*Escherichia coli*, *Salmonella typhi*, *Campylobacter* spp., *Clostridium perfringens*, *Listeria monocytogenes*, *Shigella* spp., *Enterococcus* spp., *Vibrio cholera*, etc.), protozoan (*Cryptosporidium* spp, *Giardia* spp., *Entamoeba histolytica*, *Cyclospora cayetanensis*, *Toxoplasma gondii*, etc.), parasitic worms (*Ascaris* spp., *Trichuris* spp., *Taenia* spp., *Ancylostoma* spp., *Schistosoma* spp., etc.) [37]. Improper sewage and animal waste effluent management can spread pathogenic agents into the environment, threatening the health of animals and humans.

Emerging viral pathogens of livestock exhibit marked heterogeneity in virion morphology, genome size, and nucleic acid composition, and these virological characteristics profoundly impact how diagnostic assays are selected and how accurately they perform. The priority agents considered in this review span a range from large, icosahedral symmetric dsDNA viruses like African swine fever virus to varied RNA viruses, including pleomorphic, segmented negative-sense highly pathogenic avian influenza (HPAI) H5N1 strains and smaller, spherical positive-sense species such as Japanese encephalitis and hepatitis E viruses. Consequently, this structural and genomic diversity necessitates a wide range of detection technologies. These technologies include conventional and real-time RT-PCR/qPCR, multiple isothermal amplification platforms (e.g., RT-LAMP, RPA, RAA, ERA, iiPCR, CPA), serological methods (ELISA, PRNT, VNT, IFA), and advanced molecular approaches like droplet digital PCR, high-resolution melting PCR, CRISPR-based systems, and next-generation sequencing. Table 1 provides an integrated overview of these pathogens, summarizing their morphological and genomic characteristics alongside the principal diagnostic techniques currently applied in surveillance, outbreak investigation, and routine laboratory detection.

### 3.3. Emerging Viruses in Livestock Wastewater

#### 3.3.1. Highly Pathogenic Avian Influenza (HPAI)

HPAI H5N1, a subtype of influenza A virus (IAV) within the *Orthomyxoviridae* family, poses a major international concern due to its severe effects on poultry, recent evidence in dairy cattle, and potential threat to human health [67,68]. This virus possesses a negative-sense, single-stranded RNA genome of 13.5 Kbp composed of eight distinct segments [38,39]. IAV comprises 19 hemagglutinin (HA) and 11 neuraminidase (NA) subtypes. Of these, H17N10 and H18N11 occur only in bats, while H1-H16, H19, and N1-N9 infect a wide range of avian and mammalian hosts threatening industries and human health via clade 2.3.4.4b reassortants (H5NX) [38,39,69,70]. The virus is shed heavily in the milk of infected cows, where it targets mammary tissue, and enters wastewater systems via farm effluent and municipal treatment plants after spillover from wild birds, fomites, personnel, and cow-to-cow or interspecies spread (e.g., cats, raccoons) [42,71,72,73,74,75,76,77,78]. IAV viruses, such as H5N1, exhibit moderate environmental persistence in water/wastewater, surviving for days to weeks under cool, low-pH conditions but degrading under UV, heat, or disinfectants [79,80]. Detections occur in U.S. municipal wastewater treatment plants serving dairy/poultry regions (Table 2). While the virus persists in these environments, its detection in wastewater serves as a vital sentinel tool, providing an early warning of active outbreaks within nearby livestock populations. This detection is highly relevant for surveillance, as it supports integrated monitoring strategies such as bulk milk testing, biosecurity, movement controls, and reporting, promoting collaboration and the development of future vaccines to control the spread of this zoonotic disease [81]. However, a major limitation remains the virus’s rapid evolution through reassortment and the difficulty of identifying subclinically infected cattle that continue to spread the pathogen undetected.

#### 3.3.2. Japanese Encephalitis Virus (JEV)

Japanese encephalitis virus (JEV), a mosquito-borne *Orthoflavivirus* in the *Flaviviridae* family, features a single-stranded, non-segmented positive-sense RNA genome of ~11 kb. This virus encodes a polyprotein cleaved into three structural proteins (capsid, membrane, envelope) and seven nonstructural proteins (NS1-NS5) critical for replication and immune evasion, originating from Indonesia–Malaysia with five genotypes (GI-GV, GI-III most prevalent) [45,46,82,83]. The virus is a major public health and veterinary concern due to its ability to cause severe reproductive failure in swine including mummified fetuses, stillbirths and fatal encephalitis, or permanent neurological sequelae in humans through epidemics like Japan’s 1924 outbreak [50,51,84]. It enters wastewater via piggery effluent from feces, urine, sheds, pits, and lagoons during its enzootic cycle driven by *Culex tritaeniorhynchus* mosquitoes breeding in rice fields, wading bird reservoirs (Ardeidae), and dead-end hosts like cattle, horses, humans [49,85,86]. Flaviviruses like JEV show moderate environmental persistence in water/wastewater, surviving days to weeks at cool temperatures (4–20 °C) and neutral pH conditions, but are rapidly inactivated by UV exposure, heat, salinity, or common disinfectants like chlorine [87]. Viral detection in wastewater using RT-qPCR in piggery effluent is conducted globally, often coinciding with animal/human outbreaks (Table 2). Studies targeting effluent from piggery sheds and lagoons have successfully captured viral signals, even in regions where the virus was previously thought to be restricted or dormant. It serves as an immense sentinel value for early warning, capturing pig shedding before clinical cases emerge. Surveillance integrates farm monitoring (especially summer pig testing), vector control (larvicides, adulticides), vaccination, biosecurity, and One Health reporting to curb outbreaks [86,88]. Key limitations encompass mosquito-vector dependency diluting direct shedding signals, rapid genotype emergence (e.g., GIV in Indonesia/Australia, GV in China/Korea after decades), limited persistence/stability data in farm effluents, challenges in distinguishing dead-end host contributions, and gaps in standardized global wastewater protocols amid seasonal variability [89,90,91].

#### 3.3.3. Porcine Epidemic Diarrhea Virus (PEDV)

PEDV is caused by *Alphacoronavirus porci* and is an enveloped, positive-sense, single-stranded RNA virus of the family *Coronaviridae* [92,93]. The genome size is approximately 28 kb, consisting of ORF1a and ORF1b, which encode 16 nonstructural proteins and four structural proteins—spike (S), envelope (E), membrane (M), nucleocapsid (N) and an accessory protein ORF3 [54]. It primarily targets swine, with neonatal piglets being the most susceptible host; the virus replicates in intestinal villous enterocytes, causing severe necrosis and destruction of infected cells, preventing absorption and leading to weight loss [94,95,96]. It is a critical pathogen in the global pork industry due to its high morbidity and devastating mortality rates in suckling piglets (up to 100%), with highly pathogenic G2 variants emerging in Asia and North America over the last decade [97,98,99,100,101]. PEDV sheds heavily in feces and vomit via oro-fecal or aerosol routes, and the virus enters farm wastewater and effluent through the cleaning of contaminated farrowing pens, transport vehicles, and the washing of farm equipment [96,102,103,104]. It often spreads subclinically through finishing pigs before reaching pregnant sows, which then pass it to nursing piglets [55]. PEDV shows extended stability in wastewater slurries, manure pits, coastal waters, and inflows, as confirmed by RT-qPCR and infectivity bioassays [28,105] (Table 2). RT-qPCR typically confirms the detection of viral RNA, while infectivity bioassays are used to determine whether the detected virus remains capable of causing infection (Table 2). Wastewater surveillance acts as an early warning for subclinical shedding in finishing pigs, predicting outbreaks in farrowing pens before clinical piglet mortality surges. Monitoring farm effluents for PEDV is vital for managing swine health and supporting control through biosecurity, trailer/equipment disinfection (e.g., 2.06% Clorox, 0.5% Virkon S), feed safety, movement restrictions, quarantine, and autogenous vaccines [106,107,108,109,110]. Limited data exist on Genotype 2 subgroup persistence in tropical climates (e.g., South Asia, including Karnataka’s Mandya, Bengaluru Rural, and Kolar districts of India), where highly virulent strains cause up to 100% suckling piglet mortality but lack ecological data beyond temperate regions [101]. This highlights a critical gap in understanding how these highly virulent strains behave in tropical, South Asian environments. Additional challenges include distinguishing infectious viruses from residual RNA in wastewater and the need for standardized infectivity assays beyond RT-qPCR (Table 2).

**Table 2:** Summary of detected emerging viral diseases in livestock wastewater.

Emerging Pathogen(s)	Livestock Species	Country	Wastewater Environment/ Source Type	Detection Method	Epidemiological Linkage	References
Highly pathogenic avian influenza A (H5N1) clade 2.3.4.4b *	Dairy Cattle/Poultry (indirect)	USA	Municipal wastewater serving dairy/poultry farms, raw farm effluent	Virome sequencing (Hybrid-capture sequencing); droplet digital RT-PCR (ddRT-PCR)	Signals from animal sources, particularly dairy and poultry farms, are mostly reflected in linkage across studies; detections often precede or coincide with reported livestock outbreaks and only rarely exhibit significant connection with human influenza infections. Therefore, the presence of H5N1 RNA in wastewater mostly indicates animal/ environmental source dominance, with livestock facilities and wild birds being important contributors, whereas direct human transmission signs are negligible.	[29,67,68,111,112]
Japanese encephalitis virus (JEV)	Pigs (amplifying hosts)	Australia	Piggery effluent (shed, pit, lagoon) and municipal wastewater	RT-qPCR targeting JEV RNA	JEV RNA in municipal or piggery effluent is identified by wastewater monitoring as a sensitive environmental indication of viral circulation in animal hosts, frequently before or coinciding with epidemics in humans and animals. Wastewater detection highlights the environment as a crucial reservoir and transmission mechanism in JEV epidemiology, allows for early warning, and enhances conventional monitoring.	[30,31,113]
Porcine epidemic diarrhea virus (PEDV)	Swine	USA, France	Manure pits, farm wastewater slurry, coastal waters, inflow wastewater, earthen manure storages	Reverse transcriptase quantitative PCR (RT-qPCR) for RNA, bioassays for infectivity confirmation	PEDV is frequently detected in swine manure and wastewater environments, with viral RNA often persisting for extended periods. These contaminated sources drive environmental dissemination, transmission, and occasional outbreaks among swine populations, especially when manure-handling practices spread infectious material. Studies also show PEDV can decay, but remain detectable in external environments like coastal waters, underscoring the need for effective biosecurity, manure treatment, and environmental monitoring to control disease risks.	[28,32,105]
African swine fever virus (ASFV)	Swine (domestic, feral)	UK, Australia, China, USA	Abattoir wastewater, farm drainage, environmental surface runoff	qPCR for viral DNA	Indirect transmission is a major factor in outbreaks that are made worse by human activities like animal and trash transportation. Environmental concerns may increase as a result of changes in waste management and pig production.	[33,114,115]
Hepatitis E virus (HEV) genotypes 3 and 4	Pigs, cattle, wild boars, deer, camels, goats (multiple)	South Africa, Tunisia, Cameroon	Wastewater, surface water (rivers, standpipe), piggery effluent and Abattoir	Nested RT-PCR, sequencing heminested RT-PCR, sequencing digital PCR, PacBio sequencing RT-PCR, qPCR, serology RT-PCR, sequencing	HEV genotypes 3 and 4 circulate widely in livestock such as pigs and wild boars, serving as significant reservoirs for human infections. Environmental studies detect HEV in untreated wastewater, indicating widespread viral circulation and potential contamination of water sources and food. WBS is a valuable tool for monitoring HEV spread and implementing One Health strategies addressing human, animal, and environmental health interconnectedness.	[34,64,116]

Notes: * Clade 2.3.4.4b: A phylogenetic subclade of highly pathogenic avian influenza A(H5N1) virus within the Goose/Guangdong lineage, classified according to the WHO/OIE/FAO H5N1 Evolution Working Group nomenclature system; RT-PCR: Reverse Transcription-Polymerase Chain Reaction; qPCR: quantitative Polymerase Chain Reaction; ddRT-PCR: Droplet Digital Reverse Transcription-Polymerase Chain Reaction.

#### 3.3.4. African Swine Fever Virus (ASFV)

African Swine Fever Virus (ASFV), belonging to the family *Asfarviridae*, genus *Asfivirus*, is a large, icosahedral with linear double-stranded DNA (dsDNA) genome. It replicates mainly in the cytoplasm of infected macrophages [117]. The viral genome ranges from 190 to 193 kb and contains approximately 150 ORFs [58]. It primarily affects domestic pigs (*Sus scrofa domesticus*) and wild suids, causing acute-to-chronic disease with high mortality (up to 100%) from hyperthermia, hemorrhages, bloody diarrhea, and respiratory distress [118,119,120]. First detected in Kenya (1921), genotype II re-emerged in 2007, spreading across Europe and Asia (including China, 2018); it had a devastating impact on international trade and food security due to high virulence and complex epidemiology involving soft tick vectors (*Ornithodoros* spp.) and four transmission cycles [121,122,123,124,125,126]. The virus enters wastewater systems through the shedding of infectious material in feces, urine, and blood from infected animals, as well as via contaminated farm effluent and runoff from the carcasses of wild boars or culled domestic pigs [124,127]. ASFV exhibits notable environmental stability, surviving in water >60 days at 4 °C, feces 3–8 days (37 °C), urine up to 5 days at 4 °C, and slurry post-heat treatment if incomplete; it is enhanced in cool/moist conditions but variable in warm wastewater [33,128,129]. Its complex, multi-layered structure allows it to remain infectious for months in contaminated water and years in chilled or frozen meat products [128,129]. Detection in wastewater is typically achieved through sensitive qPCR (e.g., PEG-precipitation) in SE Asian pig farm wastewater (7.5% positivity), environmental swabs (fomites, soil), aerosols, and slurries; DNA is common, but infectious virus is rare post treatment [35,59]. Wastewater serves as a sentinel for subclinical/environmental spread, enabling early outbreak detection before clinical signs like hyperthermia or hemorrhages become widespread, providing a crucial early warning to prevent catastrophic spread. In terms of surveillance relevance, wastewater testing supports early movement restrictions, tick control, strict biosecurity policies, culling, and monitoring of high-risk areas, where biological vectors such as soft ticks or wild boars are present [130,131]. Key limitations include the following: limited data on ASFV viability in tropical South Asian wastewater amid humid climates; qPCR detects DNA but struggles to differentiate infectious viruses (need viability PCR like PMA/EMA); current lack of a globally approved, safe, and effective vaccine; high culling costs; poor biosecurity in smallholder farms; gaps in wild/domestic surveillance integration [36,126,132].

#### 3.3.5. Hepatitis E Virus (HEV)

Hepatitis E virus (HEV), which belongs to the genus *Paslahepevirus* (Species: *Paslahepevirus balayani*) in the family *Hepeviridae*, is a positive-sense, single-stranded RNA (ssRNA) virus (~7.2 kbp) with ORFs encoding nonstructural proteins (ORF1), capsid (ORF2), and virion release functions (ORF3; novel ORF4 in genotype 1) [133,134,135,136]. Pigs serve as the primary livestock reservoir for significant zoonotic strains (HEV-3 and HEV-4), with importance in epidemics via fecal–oral/foodborne routes, causing hepatocellular necrosis and elevated AST/SDH [137]. HEV sheds heavily in feces, urine, and farm effluents, which are discharged into wastewater. HEV persists stably in water/wastewater as neHEV (non-enveloped) and eHEV (quasi-enveloped) forms [138,139]. Detection evidence includes HEV RNA via RT-PCR in pig feces/wastewater (China 1993; USA 1997) and swine globally; in ruminants, RT-PCR/serology was confirmed in goats, cows/milk, buffalo, sheep, camels, and deer [140,141,142,143,144,145]. Wastewater positives are reported across Asia (India outbreaks, Bangladesh pig farms), Europe (swine effluents), Africa and North America [116,146,147,148,149]. Wastewater surveillance aids livestock monitoring by tracking zoonotic HEV spillover. It supports control through biosecurity measures, including separating pigs from wildlife/ruminants, strict manure management, water quality monitoring, and avoiding undercooked meat/raw milk to interrupt fecal–oral/foodborne transmission on mixed farms. Gaps include ORF4 function (theoretical hypotheses), ruminant transmission dynamics, and surveillance challenges in low-resource areas like South Asia [150].

### 3.4. Dynamics of Emerging Viral Disease via Livestock Wastewater

**Figure 3:**
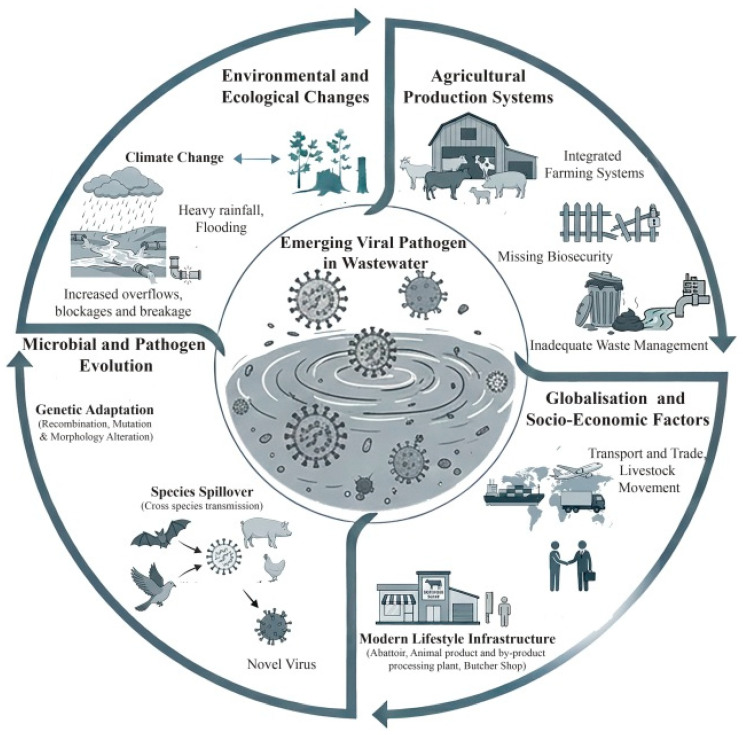
Dynamics of emerging livestock viruses in wastewater systems (conceptual framework illustrating multifactorial drivers of the emerging livestock viral pathogen) (e.g., HPAI, ASF, PEDV, HEV and ASFV). The model integrates central wastewater reservoirs with four interconnected domains: microbial evolution (genetic adaptation, species spillover), environmental changes (climate change, heavy rainfall, flooding), agricultural practices (integrated farming systems, biosecurity gaps), and socioeconomic factors (animal product trade, modern lifestyles). Bidirectional arrows represent transmission pathways amplifying zoonotic risks from livestock to human populations via fecal–oral routes).

The emergence of viral diseases in livestock arises from a complex, synergistic interplay of multifactorial drivers, as conceptualized in our multifactorial framework (Figure 3). As conceptualized in Figure 3, viral emergence via livestock wastewater is driven by four interacting variables. First, (1) environmental drivers (e.g., extreme weather, flooding) alter viral persistence and overwhelm agricultural infrastructure. This intersects with (2) agricultural variables, such as high-density farming and poor biosecurity, which dictate the volume of viral shedding into effluent. Within this matrix, (3) microbial evolution occurs, as extended survival in organic-rich slurries promotes viral adaptation. Finally, (4) socioeconomic variables, such as global trade and sanitation disparities, accelerate geographic dissemination. By systematically mapping these constituent elements, Figure 3 illustrates how localized farm effluent escalates into a global zoonotic threat, identifying key interception points for wastewater surveillance.

#### 3.4.1. Environmental and Ecological Changes

Environmental and ecological changes, such as shifts in climate patterns and natural systems, play a crucial role in boosting the emergence of viral pathogens in wastewater by stressing ecosystems and infrastructure, making it easier for viruses spread more easily. In Figure 3, it is like a chain reaction: when nature gets thrown off balance, wastewater systems cannot keep up, spilling out more untreated waste loaded with pathogens. Factors such as heavy rainfall, flooding, tsunamis and cyclones increase the severity of overflows, blockages and structural failures, thereby directly increasing the frequency and volume of untreated or partially treated sewage discharges into surrounding water bodies [151].

There will likely be more severe cases of environmental pollution as rainfall frequency and volume increase [152,153]. Increased severe weather can also lead to erosion and flooding, which can damage pipes. Wind speeds are often linked to severe weather events driven by climate change. Rainfall across complex terrain will shift accordingly, increasing upwind of hills and ranges and perhaps exacerbating some of the problems caused by rising rainfall. Due to windfall and increased debris accumulation in pump stations and screens, especially in combined systems, high winds can result in obstruction or damage. Pressurized systems that rely on electricity to transport wastewater may be indirectly impacted by wind damage to infrastructure, such as power lines [154].

In addition to this, climate change interacts with droughts and rising temperatures to change wastewater strength and treatment efficacy, favoring anaerobic conditions, corrosion, and odor generation that shorten the life and surge the likelihood of spills. Together, these environmental stresses weaken the resilience and assimilation capacity of rivers and coastal waters, allowing enteric viruses and other pathogens from wastewater to persist for longer periods, thereby increasing the potential for animal and human exposure [151]. Direct environmental effects make this threat even worse. Higher temperatures can promote viral stability and infectivity for certain enveloped viruses like IAV (H5N1) by altering lipid envelopes. Meanwhile, wild swings in pH and salinity from floods or droughts prolong the persistence of non-enveloped viruses such as HEV in aquatic environments [80,155]. For instance, warmer waters reduce UV inactivation, and boosting swine sheds more HEV into wastewater that ends up irrigating fields, facilitating zoonotic spillover to humans via contaminated wastewater [156,157]. Similarly, disrupted wetlands from flooding expand interfaces for JEV transmission, where pigs amplify the virus from mosquito vectors (*Culex* spp.) feeding on wastewater-contaminated spots, enabling cross-species jumps to humans [158]. In H5N1 cases, the HPAI virus has spilled over from wild birds (reservoirs) to poultry via diluted wastewater in flooded poultry farms or to mammals like dairy cattle via direct contact, fomites, or environmental contamination of feed/water with bird droppings, saliva, or nasal secretions, with climate-driven heat waves enhancing viral aerosol stability and airborne transmission. Together, these factors increase human–animal–vector contacts, raising cross-species transmission risks and contaminating surface/groundwater for drinking and irrigation [71,79].

#### 3.4.2. Agricultural Production Systems

Integrated livestock production and the way agricultural inputs, such as poor biosecurity on farms and inadequate manure and solid-waste management, are managed determine how efficiently viruses such as H5N1 in poultry, ASFV in pigs, PEDV in swine herds, and JEV and HEV in pig–human interfaces enter wastewater streams and environmental waters. Agricultural manure from high-density farms is frequently applied to cropland combined with other inputs like pesticides and microplastics, resulting in complex “soil viromes” where viral particles, antibiotic-resistant genes, and mobile genetic elements co-circulate and can persist in runoff after rainfall [159]. ASFV, PEDV, and H5N1 viruses can be found in sediments, irrigation water, and eventually municipal sewers due to inadequate on-farm waste management, which includes direct discharge of slurry, carcass leachate, or slaughter effluents into surface water, especially during periods of heavy rainfall and flooding [28,33,112,160]. Integrated farming systems that combine pigs, poultry, cattle and aquaculture with inadequate waste management efficiently direct viral shedding from infected herds into ponds, drainage ditches, and irrigation canals, increasing exposure for livestock, farm workers, and downstream communities.

#### 3.4.3. Microbial and Pathogen Evolution

Microbial and pathogen evolution is the inner biological engine that continually generates new or more persistent forms of emerging viral pathogens in wastewater. It links external drivers, including climate, agriculture, globalization and infrastructure, to the emergence of novel viral variants, expanded host ranges and enhanced environmental survival, thereby sustaining and renewing the risk of spillover at the human–animal–environment interface. Viral spillover is the process by which viruses cross-species barriers from reservoir hosts (often wildlife or livestock) into new susceptible hosts, such as humans, driven by genetic mutations, recombination, and ecological disruptions facilitated by environmental matrices, such as wastewater [161,162]. For example, PEDV can remain detectable and infectious for many months in manure lagoons, with evidence of ongoing viral replication in the absence of the new input, indicating that the storage environment itself enables persistence and possibly selection of fitter variants [32]. Similarly, ASFV shows notable stability in organic materials such as feces, carcass fluids and contaminated fomites, allowing prolonged circulation and the selection of strains that tolerate temperature fluctuations, desiccation or pH changes typical of wastewater and sludge pathways [115]. For enteric viruses such as HEV and JEV, the same contaminated waters are used for irrigation, aquaculture and, in water-scarce settings, even domestic purposes, providing repeated oral- or vector-mediated exposure for humans and livestock. This ecological interface allows pig-adapted or wildlife-adapted strains to cross into new hosts, establish infection and, over time, adapt genetically to more efficient replication or shedding in those hosts, further enriching the viral load entering wastewater systems.

#### 3.4.4. Globalization and Socioeconomic Factors

Globalization, trade and socioeconomic inequalities determine who is most exposed to virus-contaminated wastewater and how efficiently emerging pathogens move between regions. In many low- and middle-income countries, limited funding for water and sanitation leaves millions relying on untreated rivers, shallow wells, or poorly treated wastewater for domestic and agricultural purposes, with millions still consuming microbiologically unsafe water [163]. Poverty, low education, unequal access to safe water poor, and women’s main role in fetching water all raise the risk of infections with HEV and other water-associated viruses from contaminated water, especially in marginalized communities [164]. At the same time, global transport of live animals, feed and animal products allows pathogens such as H5N1 and ASFV to move rapidly along transport corridors and through ports linking one region’s wastewater and agricultural systems to another [165,166,167]. Additionally, modern lifestyle infrastructure, such as abattoirs, meat-processing plants, wet markets and supermarket shop environments, concentrates animal and human waste flows into shared wastewater systems [168]. When these facilities lack appropriate pre-treatment and occupational hygiene, high loads of blood, offal, intestinal contents and contaminated wash water enter municipal sewers, markedly increasing viral loads and organic strength at treatment plants and in downstream discharges.

### 3.5. Impacts of Livestock Wastewater-Related Emerging Viral Diseases

**Figure 4:**
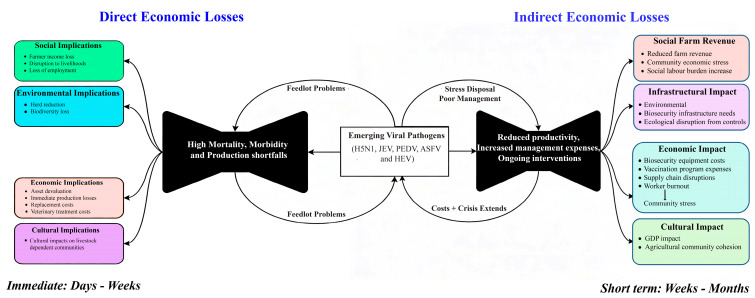
Impact pathway model of emerging viral pathogens in livestock (this bow tie diagram maps the causal link between viral pathogens (e.g., HPAI, ASF, PEDV, HEV and ASFV) and their socioeconomic consequences. The left side depicts ‘Direct Economic Losses’ occurring immediately (days to weeks), driven by high mortality and production shortfalls, with specific impacts on social, environmental, economic, and cultural sectors. The right side outlines ‘Indirect Economic Losses’ occurring over the short term (weeks to months), resulting from reduced productivity and increased management expenses, further categorized into farm revenue, infrastructural, economic, and cultural impacts.

Emerging viral diseases in livestock generate multifaceted economic impacts extending across production systems, markets, and communities through cascading pathways of direct and indirect losses. As summarized in the Impact Pathway Model (Figure 4) and the comparative analysis (Table 3), these diseases trigger a multi-dimensional cascade of losses categorized into immediate direct impacts and sustained indirect consequences. Figure 4 utilizes a ‘Bow Tie’ causal framework to systematically map the cascading economic consequences of wastewater-borne viral outbreaks. The central node represents the initial biological event (e.g., an ASFV or H5N1 outbreak). The left hemisphere categorizes Direct Economic Losses—immediate, quantifiable shocks such as mortality events, mandatory culling, and production shortfalls (e.g., $950 lost per H5N1-infected cow). The right hemisphere traces Indirect Economic Losses—long-term cascading effects that include infrastructural burdens (e.g., $11 million for PEDV biosecurity), international trade disruptions, and sociocultural impacts such as workforce burnout. By delineating these variables, Figure 4 connects the immediate virological threats detected via L-WBS to broader socioeconomic destabilization, reinforcing the value of early detection in halting the progression of outbreaks.

#### 3.5.1. Direct Economic Losses

Direct economic losses occur immediately within days to weeks following the introduction of pathogens such as H5N1, JEV, PEDV, ASFV, and HEV. These losses are primarily characterized by high mortality, morbidity, and production shortfalls. Specifically, H5N1 can result in total losses of up to $950 per cow per clinically affected cow, totaling ~$737,500 for the herd during the observation period, driven by milk reduction ($335) and substantial replacement costs ($615) [42]. Production drops are equally severe in swine, where PEDV has caused the loss of 3.7 million pigs in the US and sharp declines in piglet weaning rates in Mexico (from 9.75 to 2.43–8.07 per sow) [169,170]. The most severe production impacts are associated with ASFV, which resulted in approximately $25.9 billion in production losses in China and a 20% reduction in the total pig population in Vietnam (Table 3) [171,172]. Additionally, chronic herd losses due to the HEV impose a steady financial burden of €0.39–7.26 per pig annually [173]. Beyond production, the impact pathway model identifies immediate environmental and social implications, including reduced herd numbers, biodiversity loss, and immediate income losses for farmers (Figure 4).

#### 3.5.2. Indirect Economic Losses

In the short term (weeks to months), indirect economic losses amplify direct shocks by reducing productivity, increasing management expenses, and necessitating ongoing interventions. These costs include labor, biosecurity infrastructure, and veterinary diagnostic expenses, as seen with H5N1-related milk yield decreases of 8–14 kg/day [42]. For ASFV, the indirect impact extends to national economies, causing GDP declines of 0.4–1.8% in Vietnam and leading to the loss of up to 1.2 million jobs [171]. Substantial investments are required for disease control; for instance, Japan incurred approximately 1.18 billion JPY ($11 million USD) in biosecurity and vaccination costs following re-emergence of PEDV [174]. Similarly, HEV control measures, such as national vaccination programs, can cost up to €37 million annually in the Netherlands (Table 3) [173]. Furthermore, the model highlights broader infrastructural and economic impacts including supply-chain disruptions, worker burnout, and ecological disruptions from carcass disposal and other control measures (Figure 4).

**Table 3:** Comparative summary of emerging livestock wastewater-related viral disease—economic impact and socioeconomic implications.

Disease Name	Year of Major Outbreak	Direct Economic Losses	Indirect Economic Costs	Trade and Market Impact	Socioeconomic Implications	References
H5N1	2024–2025 (Ongoing)	$950/cow ($335 milk loss + $615 replacement); $737,500/776 cow herd; 945 kg milk loss/cow over 67 days	Labor/biosecurity; reduced productivity (8–14 kg/day lower milk); vet diagnostics	Interstate cattle movement restrictions (22+ US states); export certification barriers; raw milk diversion	$200M+ federal aid needed; worker health risks (24+ human cases); food insecurity threat	[42]
JEV	2022 (Australia: >80 pig farms affected; 60% of industry impacted, detected Feb–Mar across four states)	Australia: $215 K–$250 K USD per 1000 sows; 3–6% annual production loss. US projection: $306 M–$612 M	Not explicitly quantified (e.g., no data on surveillance, control, or long-term productivity beyond direct production losses)	Not addressed (focus on production losses; no export/trade restrictions mentioned)	Welfare impacts noted (reproductive failure, boar infertility); public health threat via zoonosis but no pig-specific socioeconomic data	[175]
PEDV	2013–2014 in the US (April–May 2013), Japan (re-emerged October 2013, peak 2014), Mexico (2013–2014)	Mortality and production drops included 3.2% reduction in US pig crop (~3.7 million pigs), 93,650 piglet deaths in southern Japan, and sharp fall in Mexican weaned piglets (from 9.75 to ~2.43–8.07 piglets/sow in weeks post-outbreak). Piglet mortality was severe with losses up to 100% in first weeks in all regions.	Increased production costs due to mortality and morbidity: US hog slaughter and retail losses up to several hundred million to billion USD annually; Japanese farms incurred biosecurity and vaccination costs totaling ~1.18 billion JPY (~US $11 million); Mexican farms had weaned piglet cost spikes to >US $100 per piglet during outbreak weeks plus higher ongoing costs post-stability.	US pork exports declined slightly (−2.7%), imports rose (+14.5%), with no major pork trade bans but some limitations on breeding swine exports. Japan experienced local supply reductions in key production regions impacting competitiveness. Mexican pork production losses translated into multi-million USD lost revenues, with economic ripple effects through input–output sectors.	Hog producers in all regions suffered uneven burden: infected farms faced losses while uninfected farms sometimes benefitted from higher prices (notably in US). Consumer prices rose, impairing consumer surplus (US losses in hundreds of millions annually). Biosecurity, vaccination, and management investments increased across all regions. Rural and pig-farming communities experienced significant economic and social strain.	[169,170,174,176]
ASFV	China: 2018 (August onset, peaked September–October) Vietnam: 2019 (February onset)	In China, production losses of US$ 25.9 billion due to mortality and US$ 8.7 million per province. Global pork prices increasing by 17–85%. In Vietnam, nearly 6 million pigs lost (20% population), sector supply drops 11–33% in traditional/ commercial.	In China, feed market disruptions; beef/poultry prices rising 1.5–6.7%; market instability recovery In Vietnam, GDP decline 0.4–1.8% (US$0.9–4.4B) and Job loss (247 K–1.2 M)	China pork imports increase by 8–32 Mt; pork supply shortage triggers price rises (11–45%) in Vietnam; global production rises 5–22 Mt outside China to offset half of losses	Household welfare in China declines 0.12–0.78% with calorie availability drops >50 kcal/day. Total loss 0.78% GDP (US$111.2B); small farms abandon production; in Vietnam, medium/large farmers lose 50–100% income; rural households hit hardest (0.3–2.2% income drop)	[171,172,177]
HEV	1983 to present (Endemic in Africa, Asia); emerging in Europe (rising cases 2005–2015)	Human health burden: chronic production loss in herds, control measures costs: €1 M (cleaning boards) to €37 M/year nationally (vaccination). Per pig: €0.39–€7.26/year.	Food safety compliance; meat inspection costs; consumer awareness campaigns	Meat export quality concerns; food import restrictions (HEV-contaminated products); market access limitations	Public health threat; consumer food safety anxiety; occupational health risk (slaughter house workers); zoonotic disease burden; limited seroprevalence awareness	[173,178]

#### 3.5.3. Trade and Market Impact

Viral outbreaks severely disrupt trade through movement restrictions, certification barriers, and supply shocks. H5N1 prompted interstate cattle movement restrictions in over 22 US states and created significant hurdles for export certification. While PEDV had a more moderate effect on US trade, reducing pork exports by 133.855 million pounds (2.68%) in 2014 compared to 2013 levels, imports rose 127.895 million pounds (14.54%), and it significantly limited breeding swine movement [169]. Market volatility is most pronounced with ASFV, which triggered pork price rises of 11–45% in Vietnam [171]; meanwhile, China’s pork imports exceeded one-tenth of the total world trade from 2011 and reached 15% of domestic consumption by 2015, though it nearly maintained self-sufficiency until then [179]. Finally, HEV affects trade through food safety compliance requirements and concerns about meat export quality.

#### 3.5.4. Socioeconomic Implications

Emerging livestock diseases impose profound socioeconomic burdens, particularly on vulnerable rural communities, by disrupting livelihoods, undermining food security, and amplifying public health risks. Individual farmer losses frequently precede and drive macroeconomic impacts, as evidenced across geographic and production systems. Uncontrolled disease transmission inflicts catastrophic losses on small-scale producers with minimal asset recovery capacity. In Vietnam, ASF outbreaks triggered income losses of 50–100% among medium- and large-scale swine farmers, while dairy farmers in the United States affected by HPAI H5N1 (2024–2025) experienced pronounced milk production losses of 945 kg per clinically affected cow over 67 days, resulting in economic losses of approximately $950 per affected animal and compounded herd losses exceeding $737,500, with recovery timelines extending beyond four months and elevated risks of premature herd removal [171,180]. In Haiti, ASF-induced pig population collapse (71% decline) and reduced meat-processing (43% drop) catalyzed broader household economic shocks: affected families saw primary school enrolment fall by 14%, food insecurity reached 47%, and rural out-migration accelerated such that the rural population share contracted by 15% within two decades [181]. When a disease hits a farm, the damage does not stop there; it creates a domino effect that quickly raises food prices and hurts the entire national economy. In China, the world’s largest pork producer, ASF, generated a documented 0.78% contraction in gross domestic product (GDP) in 2019, with worst-case scenarios projecting GDP declines of 1.4–2.07% [172]. Direct costs across China and neighboring countries are estimated at $130 billion [182]. These shocks disrupt dietary intake in vulnerable populations such as China, which reduced per capita calorie availability by over 50 kcal per day, which is a critical threshold for nutritionally vulnerable low-income households [177]. Similarly, in the United States, PEDV generated net annual welfare losses of US$900 million to US$1.8 billion through reduced pork supply, higher feed costs for competing proteins, and consumer surplus losses of US$300–600 million [169,183]. Public health threats also represent a major socioeconomic concern; H5N1 and HEV pose zoonotic risks and occupational hazards for slaughterhouse workers, leading to consumer food safety anxiety and significant community stress. For instance, HEV seroprevalence in swine workers (47%) and slaughterhouse personnel substantially exceeds general population levels (26.1%), indicating direct animal-to-human transmission risk through occupational exposure and inadequate hygiene practices [184].

### 3.6. Wastewater Surveillance: A Technical Execution of One Health for Spillover Mitigation

The emergence and re-emergence of EIDs are fundamentally linked to the animal–human–environment interface [185], which is a premise central to this research. By providing continuous, ecosystem-level data, WBS systems move beyond the siloed monitoring of human or animal populations in isolation. The detection of specific pathogens, especially viral pathogens and antimicrobial resistance genes (ARGs) in livestock effluent, provides high-resolution, community-level data that creates a quantifiable environmental risk profile, bridging the gap between veterinary medicine, environmental health, and human public health surveillance [186,187,188,189].

The most significant advantage of this method is its ability to provide quick, practical warnings before the clinical disease presentation is visible in either human or animal populations [20]. This advanced warning capability transforms conventional passive disease reporting into a sophisticated proactive defensive system [190]. A powerful illustration is found in the surveillance of agricultural coronaviruses [191]. For instance, the successful detection of the swine acute diarrhea syndrome coronavirus (SADS-CoV) in piggery wastewater and fecal samples provided researchers with an antecedent warning of a potential zoonotic alphacoronavirus threat, allowing for rapid characterization of the emerging pathogen and early risk assessment [192,193,194]. Furthermore, surveillance targeting high-consequence zoonoses, such as HPAI (H5N1) or zoonotic arboviruses such as JEV in poultry or swine effluent, establishes an immediate environmental trigger [12]. This signal permits veterinary and public health authorities to deploy pre-emptive interventions, such as heightened farm biosecurity, targeted vaccination campaigns for at-risk livestock or local communities, and the judicious application of livestock movement restrictions. These actions are indispensable for drastically reducing the risk of zoonotic spillover and mitigating its catastrophic consequences.

Wastewater surveillance fundamentally strengthens the environmental pillar of the One Health mandate by extending beyond single-pathogen detection to encompass the surveillance of ARGs in agricultural runoff [195,196,197]. The abundance of ARGs in livestock waste is higher than in other reservoirs [198]. ARGs are frequently detected in livestock wastes (including solids used for manure, wastewater, and lagoon slurry) and sediments at much higher levels (up to 28,000 times) than in background soil or upstream water [199,200,201]. Monitoring the concentration and diversity of ARGs in swine and cattle manure and lagoons offers a proxy for assessing the efficacy of on-farm AMR stewardship programs and the safety of manure treatment protocols prior to land application [202]. This environmental assessment is crucial for mitigating the global health crisis stemming from the dissemination of resistance mechanisms into the wider ecological network and food system. For managing common endemic diseases, such as PEDV, wastewater monitoring provides objective, quantitative assurance regarding the effectiveness of farm decontamination procedures and biosecurity measures. This allows farmers to confirm viral elimination from the environment and manage subsequent production cycles with greater certainty [28].

In summary, WBS transforms passive surveillance disease reporting into an active, prospective defensive mechanism. This evidence-based, One Health-driven strategy is indispensable for curtailing (EID) outbreaks and mitigating the catastrophic economic and human health ramifications that follow uncontrolled zoonotic events. The continuous, integrated environmental data stream generated by WBS fundamentally shifts the public health paradigm from reactive to preventive.

### 3.7. Challenges and Technological Standardization

While the utility of WBS as a prospective biosentinel is conceptually straightforward, its wide-scale implementation across diverse agricultural landscapes faces several key challenges that necessitate standardization and technological refinement. A fundamental methodological challenge is the profound physicochemical discrepancy between human domestic sewage and animal agricultural waste. Unlike the relatively consistent matrix of municipal sewage, agricultural effluent is notoriously complex and heterogeneous, varying drastically in solid content, chemical composition, and flow rate based on the animal species, housing system, and manure management strategy [203,204]. Crucially, livestock wastewater is heavily burdened with dense organic matter and suspended solids, undigested or partially digested plant materials such as dietary grains and seed husks in swine/cattle effluent, and high-fiber particulate content [205,206,207,208].

These solid constituents impose significant physical and biochemical barriers during sample processing. Physically, high-fiber matrices often cause severe membrane fouling during filtration and impede efficient viral elution. Biochemically, the high concentration of plant fibers and animal feces introduces potent PCR inhibitors—including complex dietary polysaccharides, humic acids, and bile salts [209,210,211]. These inhibitors can severely attenuate the amplification efficiency of molecular diagnostic tools such as RT-qPCR or ddPCR, often resulting in false-negative results or significant underestimation of viral load [207]. Inadequate removal of these inhibitors directly compromises the sensitivity and stability of molecular surveillance [212]. Consequently, the translating of domestic wastewater protocols directly to agricultural settings is often inefficient, affecting viral concentration and nucleic acid extraction efficiency, potentially introducing biases into quantitative metrics, and complicating comparisons across farms or regions [213]. To overcome these matrix-induced biases, optimizing virus concentration is a critical step for detecting low concentrations of viruses and their variants in wastewater. The most used methods were polyethylene glycol (PEG) precipitation, ultrafiltration, electro-negative membrane filtration, ultracentrifugation, aluminum hydroxide adsorption precipitation, aluminum chloride precipitation, and skimmed milk (SM) flocculation [214,215]. Establishing standardized, matrix-specific protocols that incorporate enhanced solid–liquid separation and robust inhibitor-removal technologies tailored for fiber-dense animal waste is an urgent necessity [213,216]. For instance, optimized protocols such as beef extract-enhanced polyethylene glycol (BE-PEG) precipitation (using 8% PEG-8000 with 0.3 M NaCl, pH-adjusted centrifugation, and overnight 4 °C incubation) and Amicon ultrafiltration followed by virus quantification using quantitative PCR (qPCR) achieve high viral recovery rates (10–14%) across diverse matrices, outperforming standard PEG methods, especially in high-solid manure effluents. The Amicon method is suitable for small sample volumes with high ammonium levels and, therefore, should be used when abundant viruses are quantified, whereas the precipitation method may be used for early detection when levels are low and for rare pathogens [217].

Furthermore, while quantitative polymerase chain reaction qPCR offers rapid, high-throughput pathogen detection, future WBS efforts must increasingly incorporate next-generation sequencing NGS, particularly shotgun metagenomics. These advanced techniques provide the genomic resolution necessary to track precise phylogenetic lineages, identify novel resistance mechanisms, and differentiate clinically relevant strains from less virulent environmental background populations [218,219]. Overcoming these analytical hurdles through unified sampling protocols (e.g., standardized 40 mL aliquots with PEG-8000 centrifugation at 12,000× *g* for 2 h) and validated concentration/extraction methods is crucial for translating raw WBS data into globally comparable veterinary and public health intelligence [204,220]. Inter-laboratory comparison studies may greatly help identify discrepancies and errors in analytical results arising from various factors or identify the most significant sources of variation. The diverse approaches still employed across various steps in WES workflows highlight the ongoing challenges of harmonization, underscoring the need for standardized methodologies and reference materials [221]. Inter-laboratory ring tests, such as the 2025 Lombardy initiative across veterinary labs using harmonized qPCR targets (N1/N3/ORF1ab) and NIST RNA standards, reduce pre-analytical variability and support livestock applications, such as H5N1 dairy surveillance [221,222]. Such validations pinpoint methodological discrepancies and emphasize the use of reference materials for workflow harmonization.

### 3.8. Evidence for Early-Warning Potential of Wastewater Surveillance

Extensive research demonstrates that wastewater surveillance can serve as an early-warning system, detecting viruses in the environment well before clinical outbreaks are formally recognized. During the COVID-19 pandemic, wastewater monitoring consistently detected SARS-CoV-2 spikes in the virus days to weeks before a noticeable increase in clinical cases, demonstrating its value as a powerful and effective early-warning tool for the public health [223,224,225,226]. For instance, temporal analyses across diverse urban catchments demonstrated that viral RNA signals in municipal sewage preceded confirmed clinical case reports by six to eight days, effectively capturing the initial onset of outbreaks [223,224]. This predictive lead time is fundamentally driven by the immediate detection of fecal viral shedding from asymptomatic and pre-symptomatic individuals, which circumvents the inherent testing bottlenecks and reporting delays of traditional clinical diagnostics [226]. Consequently, quantitative wastewater models have been shown to predict actual community infection caseloads up to two orders of magnitude higher than officially reported figures, highlighting the critical role of environmental monitoring in revealing silent viral circulation [225]. Beyond SARS-CoV-2, wastewater surveillance has historically proven essential for the early detection of circulating pathogens such as poliovirus and antimicrobial resistance genes, further validating its broader utility as a proactive, population-level epidemiological indicator [202,208,227,228]. Building upon these public health successes, recent evidence demonstrates that this predictive capacity translates effectively to veterinary and agricultural sectors. A prominent example occurred during the 2024 highly pathogenic avian influenza A(H5N1) outbreak in the United States, where wastewater monitoring networks successfully detected significant levels of the H5 influenza subtype in municipal treatment plants serving agricultural communities [67,111]. Crucially, retrospective analyses by the WastewaterSCAN consortium revealed that spikes in H5 viral RNA were detected in Texas wastewater treatment plants weeks prior to the first official reports of the disease in local dairy cattle [67].

Despite the conceptual promise of L-WBS, practical implementation faces substantial barriers. While advanced molecular diagnostics like next-generation sequencing (NGS) and droplet digital PCR (ddPCR) offer unparalleled sensitivity, their deployment in agricultural settings is severely constrained by high capital costs, complex bioinformatic requirements, and a critical shortage of trained personnel in rural areas [229,230]. Relying exclusively on these high-throughput technologies creates a significant accessibility gap, particularly in low- and middle-income countries (LMICs). Furthermore, while the current literature frequently promotes L-WBS as an early-warning system, this capability is largely extrapolated from retrospective studies. Because retrospective analyses benefit from prior knowledge of clinical outbreak timelines, they risk overestimating actual predictive accuracy [231]. Until robust, prospective cohort studies are conducted in real time across diverse agricultural settings, the definitive predictive lead time of L-WBS remains an empirical hypothesis requiring rigorous validation [12].

### 3.9. Policy Integration and Economic Imperatives

The fundamental shift from reactive clinical diagnostics to proactive environmental monitoring yields significant policy and economic benefits that go beyond simple disease management. WBS provides the objective, longitudinal evidence required to support risk-based regulatory decisions concerning AMR mitigation and the implementation of One Health policies, as evidenced by the EU’s One Health Action Plan (2017–2025) which integrates wastewater surveillance data into zoonotic risk assessment frameworks and the U.S. CDC’s National Wastewater Surveillance System (NWSS), which now covers 1000+ sites monitoring 160 million Americans for 20+ pathogens including poliovirus and influenza [232,233,234,235]. Economically, the temporal advantage offered by pre-symptomatic detection of high-consequence pathogens—such as PEDV or HPAI H5N1 in dairy cattle—allows for targeted and rapid intervention, dramatically reducing the potential for catastrophic economic losses associated with mass culling, trade restrictions, and prolonged recovery periods [8,236]. The 2013–2014 PEDV outbreak costs the U.S. swine industry $900 million–$1.8 billion annually, while HPAI H5N1 (2024–2025) inflicted $950 per affected dairy cow ($737,500 per 776-head herd), with nationwide impacts across 875+ herds and 4+ month recovery periods [180,183].

Moreover, by providing farm-specific, verifiable metrics on the efficacy of biosecurity and manure treatment protocols, WBS incentivizes producers to invest in superior stewardship programs [237,238]. This objective environmental data can be integrated into established national and international food safety certification schemes such as GlobalG.A.P. standards [239] and USDA Process Verified Programs, offering transparent, auditable measures of farm hygiene and pathogen control that support premium pricing and market access for compliant producers [240,241].

Ultimately, WBS represents a cost-effective, continuous intelligence gathering system that protects both food security and underpins the strategic defense against the escalating threat of zoonotic spillover events [242,243,244]. WBS provides a scalable, cost-effective alternative to individual clinical diagnostics, enabling routine population-level monitoring without the logistical burden and high costs associated with swab-based testing infrastructure [224]. This continuous surveillance capability transforms disease management from episodic crisis response to systematic risk mitigation, providing actionable intelligence for both farm-level biosecurity and national food security planning [8,233].

### 3.10. Knowledge Gap, Future Directions and Recommendation

Emerging livestock viruses with zoonotic potential, such as ASFV, PEDV, JEV and H5N1, are prime candidates for detection in wastewater and sewage due to their fecal shedding and environmental persistence in farm effluents [59,245,246]. These pathogens can enter sewage systems via urine, feces and manure from infected pigs, cattle, and other livestock, particularly in intensive production systems where waste management and biosecurity practices may be suboptimal. Once introduced, such viruses pose a risk of dissemination through water bodies, irrigation systems, and downstream food chains, thereby amplifying opportunities for interspecies transmission and zoonotic spillover.

In contrast, several emerging livestock viruses, including vector-borne pathogens such as bluetongue virus (BTV), lumpy skin disease virus (LSDV), and Rift Valley fever virus (RVFT), are rarely detected through conventional wastewater surveillance approaches because their transmission depends primarily on arthropod vectors rather than fecal or urinary shedding [247,248]. This highlights a critical surveillance gap, whereby pathogens of substantial veterinary and public health importance may evade detection within existing WBS frameworks. Nonetheless, the high viral loads achieved during epizootics, combined with the relative stability of some viruses in organic-rich wastewater matrices, suggest that tailored sampling strategies, targeted molecular assays, and integration with vector and environmental surveillance could substantially enhance early-warning capacity.

Addressing these knowledge gaps will require a more nuanced, pathogen-specific application of WBS that accounts for viral biology, shedding dynamics, transmission routes, and environmental stability. Integrating wastewater surveillance with traditional veterinary diagnostics, syndromic reporting, and vector surveillance within a One Health framework is essential to improve preparedness, risk assessment, and outbreak mitigation at the livestock–human–environment interface.

## 4. Conclusions

The increasing frequency and geographic spread of EID outbreaks in livestock populations, along with their significant zoonotic potential, necessitate a fundamental rethinking of disease surveillance strategies. This systematic review provides compelling evidence that WBS represents a transformative advance, shifting disease monitoring from a predominantly reactive, clinically driven model to a proactive and anticipatory system rooted in environmental epidemiology. By capturing population-level signals of viral circulation independent of clinical presentation, WBS offers a powerful complementary tool for early detection, situational awareness, and risk forecasting of livestock-associated pathogens. When strategically integrated with veterinary surveillance, environmental monitoring, and public health systems, WBS can substantially strengthen One Health surveillance architectures. Ultimately, to realize the promise of L-WBS as a global frontline defense, policy implementation must adopt differentiated, context-specific pathways. In high-income countries with established veterinary infrastructure, policies should prioritize the integrating of advanced technologies, such as next-generation sequencing (NGS) and environmental metagenomics, and establish real-time data-sharing pipelines between agricultural effluent monitoring stations and national veterinary regulators. Conversely, in low- and middle-income countries (LMICs) with fragmented infrastructure and financial constraints, operational priorities must focus on fundamental capacity building. Implementation should target low-cost RT-qPCR screening at high-risk convergence points (e.g., abattoirs and live-animal markets), supported by international subsidies for mobile diagnostic training. By adopting this tiered, financially viable approach, global stakeholders can establish an equitable and resilient L-WBS early-warning infrastructure.

## Figures and Tables

**Table 1:** Morphology, genomic characteristics, disease impact, and detection techniques of emerging viruses related to livestock wastewater.

Virus	Family	Genus	Species	Shape/Morphology	Genome (Size and Organization)	Targeted Livestock	First Found (Location/Year)	Disease Consequence	Detection Methods	References
H5N1	*Orthomyxoviridae*	*Alphainfluenzavirus*	*Influenza* *A virus*	Pleomorphic, enveloped	13.5 Kbp; ssRNA (−) virus, segmented	Poultry (chickens, ducks, turkeys), dairy cattle in recent outbreaks (2024)	First detected as A/goose/Guangdong/1/1996 in domestic waterfowl (geese) in Guangdong Province, southern China (1996); precursor of the current Goose/Guangdong H5N1 lineage that spread across Asia, Europe, Africa and the Americas. Recent dairy infections in the US (2024)	Severe respiratory disease, high mortality in poultry (up to 100%), significant milk production loss (900+ kg/cow over 60 days) and economic losses (~$950 per cow)	ELISA, RT-PCR, qRT-PCR, RPA, RT-LAMP, FET, NASB, SPR, NGS	[38,39,40,41,42,43,44]
JEV	*Flaviviridae*	*Orthoflavivirus*	*Orthoflavivirus japonicum*	Spherical, enveloped	11 kbp; ssRNA (+) virus, non-segmented	Swine (primary), horses, cattle (occasionally)	JEV epidemics first recognized in Japan in 1871; first major documented human outbreak in 1924. JEV (Nakayama strain) first isolated from a fatal human case in Japan in 1935. The virus is now endemic across much of East and Southeast Asia and has recently expanded into Australia	Reproductive failure in swine (50–70% losses), high mortality in suckling piglets (near 100% in naive). Stillbirths and mummified fetuses; in cattle, usually sporadic neurologic signs and occasional reproductive loss	PRNT, CFT, LFA, IFA/IIFT, RT-PCR, RT-LAMP, ELISA (IgM/IgG), Biocensors, NGS	[45,46,47,48,49,50,51]
PEDV	*Coronaviridae*	*Alphacoronavirus*	*Alphacoronavirus porci*	Pleomorphic, enveloped with spikes	28 Kbp; ssRNA (+) virus, non-segmented	Domestic pigs (all ages), with suckling and neonatal piglets being most severely affected; no other livestock species are known natural hosts	First recognized in UK (1971) and first isolated in Belgium in 1978, spread to Europe and Asia by 2013, as well as USA (May 2013 in Iowa)	Acute severe diarrhea and vomiting in piglets, mortality up to 100% in neonates, significant morbidity and economic losses to swine industry	EM, ISH, RT-PCR, nRT-PCR, RT-qPCR, RT-LAMP, RT-PSR, RPA, RAA, ERA, iiPCR, CPA, IC, ddPCR, FMIA, NanoPCR, ELISA (IgG/IgA), VNT, IFA, Biocensors	[52,53,54,55]
ASFV	*Asfarviridae*	*Asfivirus*	*Asfivirus haemorrhagiae*	Large icosahedral, double-enveloped	170–194 Kbp; dsDNA virus, linear	Domestic pigs, wild boar and feral pigs (*Sus scrofa*); African wild suids (warthog, bushpig, giant forest hog) act as reservoir hosts with minimal clinical disease	Kenya (endemic in sub-Saharan Africa, Madagascar, in 1921). Historically confined to sub-Saharan Africa, later introduced to Europe (e.g., Sardinia) and the Caucasus (Georgia 2007), from which the genotype II pandemic spread across Europe and Asia	Haemorrhagic fever with near 100% mortality in acute form, massive economic losses (~80,000+ pigs died in Georgia 2007). High virulence	PMA-qPCR, RT-qPCR, ddPCR, iiPCR, RPA, LAMP, RAA, HCR, CLIA, PIC, CRISPR-Cas12a, Chimeric DNA/LNA-based biosensor	[56,57,58,59,60,61]
HEV	*Hepeviridae*	*Paslahepevirus*	*Paslahepevirus balayani*	Spherical, non-enveloped virions in feces (~27–34 nm); quasi-enveloped particles in blood	7.2 Kbp; ssRNA (+) virus	Domestic pigs and wild boar are the principal livestock reservoirs for zoonotic genotypes 3 and 4; infection also documented in farmed deer, rabbits, small ruminants, camels and other species	First animal strain identified in pigs in the US (1997), recognized in humans since early 1950s in India	In pigs, infection is usually subclinical, with no overt hepatitis; occasional reports suggest mild growth retardation or suboptimal performance at herd level. However, very high seroprevalence and frequent fecal shedding make pigs a critical reservoir for food-borne and occupational zoonotic transmission to humans (acute hepatitis)	RT-PCR, RT-qPCR, Droplet digital RT-PCR, RT-LAMP, RT-RPA, Immuno-peroxidase monolayer assay, Competitive ELISA	[62,63,64,65,66]

Notes: ssRNA: single-stranded RNA; dsDNA: double-stranded DNA; −: Negative sense; +: Positive sense; ELISA (IgG/IgA): Enzyme-Linked Immunosorbent Assay for Immunoglobulin G/A Detection; c-ELISA: Competitive-ELISA; PRNT: plaque reduction neutralization test; LFA: Lateral Flow Assay; LFD: Lateral Flow Dipstick Assay; FMIA: Fluorescent Microsphere Immunoassay; IHC: Immuno-histochemistry; IFA: Immuno-fluorescence Assay (IIFT: Indirect Immuno-fluorescence Assay); IPMA: Immuno-peroxidase monolayer assay; IC: Immuno-chromatography Assay (LFA: Lateral Flow Assay); VNT: Virus Neutralization Test; CFT: Complement Fixation Test; FM-ICA: Fluorescent Microsphere-based Immuno-Chromatographic Assay; CLIA: Chemi-luminescent immunoassay; AGID: Agar gel immunodiffusion; CIE: Counter-immuno-electrophoresis; MIA: Microsphere Immunoassay; RT-PCR: Reverse Transcription-Polymerase Chain Reaction; nRT-PCR: Nested Reverse Transcription-PCR; RT-qPCR: Reverse Transcription-quantitative PCR; PMA-qPCR: Propidium Monoazide qPCR; RT-LAMP: Reverse Transcription-Loop-Mediated Isothermal Amplification; RT-PSR: Reverse Transcription Polymerase Spiral Reaction; RPA: Recombinase Polymerase Amplification; RAA: Recombinase-Aided Amplification; P*f* Ago: *Pyrococcus furiosus* Argonaute; ERA: Enzymatic Recombinase Amplification; iiPCR: Insulated Isothermal PCR; CPA: Cross-Priming Amplification; ddPCR: Droplet Digital PCR; NanoPCR: Nanoparticle-Assisted PCR; HRM PCR: High-resolution melting PCR; ISH: In situ Hybridization; HCR: Hybridization chain reaction; NASB: Nuclear Acid Sequence-Based Amplification; NGS: next-generation sequencing; EM: Electron Microscopy; Biosensors (SPR: Surface Plasmon Resonance; FET: Field-Effect Transistor; PIC: Photonic Integrated Circuit; Chimeric DNA/LNA-based biosensor).

## Data Availability

No new data were created.

## Supplementary Materials

The following supporting information can be downloaded at https://www.mdpi.com/article/10.3390/v18030385/s1, Table S1: PRISMA checklist, Table S2: Risk-of-bias assessment.
